# Freely available artificial intelligence for pelvic lymph node metastases in PSMA PET-CT that performs on par with nuclear medicine physicians

**DOI:** 10.1007/s00259-022-05806-9

**Published:** 2022-04-27

**Authors:** Elin Trägårdh, Olof Enqvist, Johannes Ulén, Erland Hvittfeldt, Sabine Garpered, Sarah Lindgren Belal, Anders Bjartell, Lars Edenbrandt

**Affiliations:** 1 Department of Translational Medicine and Wallenberg Centre of Molecular Medicine, Lund University, Malmö, Sweden; 2 Department of Clinical Physiology and Nuclear Medicine, Skåne University Hospital, Carl Bertil Laurells gata 9, 205 02 Malmö, Sweden; 3 Eigenvision AB, Malmö, Sweden; 4 Department of Electrical Engineering, Chalmers University of Technology, Gothenburg, Sweden; 5 Department of Surgery, Skåne University Hospital, Malmö, Sweden; 6 Department of Urology, Skåne University Hospital and Lund University, Malmö, Sweden; 7 Department of Clinical Physiology, Region Västra Götaland, Sahlgrenska University Hospital, Gothenburg, Sweden; 8 Department of Molecular and Clinical Medicine, Institute of Medicine, Sahlgrenska Academy, University of Gothenburg, Gothenburg, Sweden

**Keywords:** Deep learning, Convolutional neural network, PSMA, Artificial intelligence, Prostate cancer

## Abstract

**Purpose:**

The aim of this study was to develop and validate an artificial intelligence (AI)-based method using convolutional neural networks (CNNs) for the detection of pelvic lymph node metastases in scans obtained using [^18^F]PSMA-1007 positron emission tomography-computed tomography (PET-CT) from patients with high-risk prostate cancer. The second goal was to make the AI-based method available to other researchers.

**Methods:**

[^18^F]PSMA PET-CT scans were collected from 211 patients. Suspected pelvic lymph node metastases were marked by three independent readers. A CNN was developed and trained on a training and validation group of 161 of the patients. The performance of the AI method and the inter-observer agreement between the three readers were assessed in a separate test group of 50 patients.

**Results:**

The sensitivity of the AI method for detecting pelvic lymph node metastases was 82%, and the corresponding sensitivity for the human readers was 77% on average. The average number of false positives was 1.8 per patient. A total of 5–17 false negative lesions in the whole cohort were found, depending on which reader was used as a reference. The method is available for researchers at www.recomia.org.

**Conclusion:**

This study shows that AI can obtain a sensitivity on par with that of physicians with a reasonable number of false positives. The difficulty in achieving high inter-observer sensitivity emphasizes the need for automated methods. On the road to qualifying AI tools for clinical use, independent validation is critical and allows performance to be assessed in studies from different hospitals. Therefore, we have made our AI tool freely available to other researchers.

## Introduction

Prostate cancer is the most common malignancy in men, and correct staging of the disease is important for the selection of appropriate treatment strategies. Prostate-specific membrane antigen (PSMA)-ligand positron emission tomography-computed tomography (PET-CT) imaging has recently been introduced for primary staging of high-risk prostate cancer patients with biochemical recurrence. The method has been shown to be more sensitive and accurate than the conventional imaging standard of CT and bone scan [1–4].

The interpretation of PET-CT scans relies heavily on visual analysis, where suspected malignant lesions are detected by a nuclear medicine physician or radiologist. Despite efforts to standardize the interpretation of PSMA PET-CT scans [5–7], both intra- and inter-observer disagreements have been found, even in single-centre studies [8, 9]. Therefore, there is an unmet need to interpret PSMA PET-CT scans objectively to increase reproducibility.

Artificial intelligence (AI) can be trained to help with the detection of metastases. A few attempts to identify or quantify tumours in PSMA PET-CT scans have been made [10–14]. Recently, automated prostate molecular imaging standardized evaluation (aPROMISE) software was developed. This CE-marked and FDA-approved software uses deep learning technology to segment organs in CT scans and classical image analysis methods to detect tumours in [^18^F]DCFPyL PET-CT scans [10]. The software has high overall high sensitivity in detecting potential lesions, but there are a large number of false positive lesions per patient, which limits the efficiency for interpreting physicians. Thus, the aim of the present study was to develop and validate an AI-based tool for the detection of pelvic lymph node metastases in [^18^F]PSMA PET-CT. A secondary aim was to make the AI-based method freely available to other researchers.

## Material and methods

### Patients and imaging

The study included 211 patients who were referred for clinically indicated [^18^F]PSMA-1007 PET-CT for initial staging due to high-risk prostate cancer at Skåne University Hospital, Lund and Malmö, Sweden, from December 2019 to March 2020. Patients were administered 4 MBq/kg [^18^F]PSMA-1007, and after 120 min, they were scanned on a Discovery MI PET-CT (GE Healthcare, Milwaukee, WI). The patients were scanned from the mid-thigh to the base of the skull.

Scans were acquired for 2 min/bed position. The PET scans were reconstructed using a block-sequential regularization expectation maximization algorithm (Q.Clear; GE Healthcare, Milwaukee, WI), including time-of-flight and point spread function modelling with a 256 × 256 matrix (pixel size 2.7 × 2.7 mm^2^, slice thickness 2.8 mm) and a beta factor of 800 [15]. CT scans were acquired for attenuation correction of the PET scans and anatomic correlation. A diagnostic CT with intravenous and oral contrast was performed.

Tube current modulation was applied by adjusting the tube current for each individual with a noise index of 37.5 and a tube voltage of 100 kV, and the slice thickness was 0.625 mm. The CT used for attenuation correction was acquired in the late venous phase, and an adaptive statistical iterative reconstruction technique was applied. The study was conducted according to the principles expressed in the Declaration of Helsinki and approved by the local research ethics committee at Lund University (#2016/417 and #2018/753). All patients provided written informed consent.

### Manual segmentations for training

Three physicians (readers A, B, and C) independently segmented suspected lymph node metastases below the aortic bifurcation (defined as “pelvic” in this article) in the [^18^F]PSMA PET-CT scans. The readers have substantial experience in PET-CT reading (two with > 10 years and one with 7 years of PET-CT experience, and all with > 3 years of [^18^F]PSMA PET-CT experience). The cloud-based annotation platform RECOMIA (https://www.recomia.org) was used for the manual segmentations and includes basic display features for PET-CT scans and segmentation tools [16]. Lymph nodes were graded according to E-PSMA guidelines [17]. Grade 1–2 lymph nodes were considered benign, while grade 4–5 were considered pathological. Grade 3 was considered pathological when deviating from known patterns of unspecific uptake such as low–intermediate uptake along the external iliac vessels. From the full set, 50 scans with three manual segmentations each were used as a test set. The remaining 161 scans were divided into a training set (125 scans) and a validation/tuning set (36 scans).

### AI tool

The AI tool is based on a UNet3D convolutional neural network (CNN) [18] that is trained to classify each pixel as either background or lymph node metastasis. Similar to a previous study [19], the input to the CNN consists of a CT image, standardized uptake value (SUV) image, and an organ mask, which is created using an organ segmentation network [16]. The organ mask has three channels, which encode (1) the prostate and urinary bladder, (2) gastrointestinal tract, and (3) any bone.

### Training the network

A convolutional neural network is trained by feeding annotated image patches to the network, while trying to minimize a loss function measuring the difference between the network output and the manual annotations. In this work, the categorical cross entropy loss was used and minimization was performed using a variant of stochastic gradient descent. Each training batch consists of 8 patches with dimensions of 100 × 100 × 100 pixels, which are chosen such that 60% are centred on a pixel marked as lymph node metastasis. Pixels above the aortic bifurcation are ignored. An epoch is defined as 2500 batches for the training set and 500 batches for the validation set.

As mentioned, there are three independent manual segmentations for each study. During training, these are essentially treated as three different images. However, to favour high sensitivity, no loss is assigned to background pixels if one of the other readers has marked them as metastasis.

#### Hard examples

After 50 epochs or when the validation loss has not improved for 10 epochs, the training is halted, and the current model is used to generate a segmentation for each image. From this segmentation, up to 20,000 incorrectly labelled pixels are marked as *hard examples*. The training is restarted but with 20% of the samples chosen from among the hard examples. This procedure is repeated four times.

#### Implementation details

Optimization was performed with the Adam method [20] with Nesterov momentum. To avoid overfitting, we used early stopping, dropout with a rate of 25%, and l2 regularization with a weight of 0.001. Input patches were augmented using rotation of − 0.15 to 0.15 radians, scaling of − 10 to 10%, and intensity shifts of − 100 to + 100 HU for the CT image and − 0.5 to + 0.5 for the SUV image.

### Post-processing

Pixels above the aortic bifurcation are automatically set to background using the same organ segmentation method [16] that was used to create the input mask.

### Statistical analysis

**Fig. 1:**
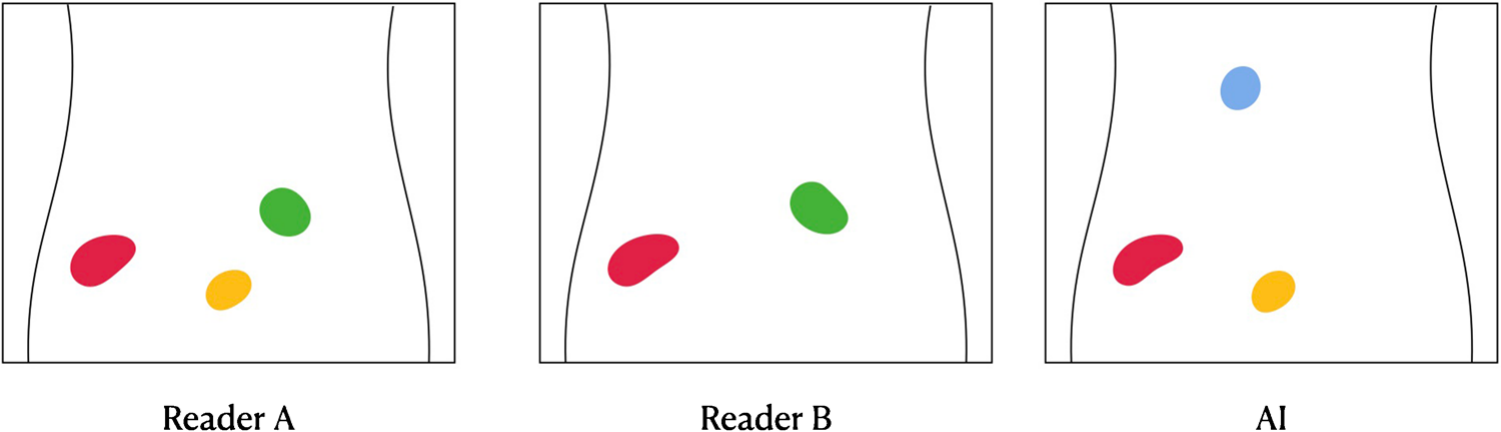
Example of how sensitivity was calculated using different readers as a reference. In this case, reader B detects 2/3 of the lesions marked by reader A, giving a sensitivity of 67%, whereas reader A detects both lesions marked by reader B, giving a sensitivity of 100%. Similarly, the AI model has a sensitivity of 67% with reader A as a reference and 50% with reader B as a reference

True positive lesions for AI or a reader, respectively, were defined as either full or partial segmentation overlap with another reader used as reference, or else they were considered false negative. Lesions detected by AI or a reader without segmentation overlap with another reader used as reference were considered false positive. The sensitivity was calculated as the proportion of suspected lymph node metastases detected by the AI method or a reader out of those detected by a reader used as reference. Figure 1 shows an example, where reader A is first used as a reference, followed by reader B, giving different values of sensitivity. The positive predictive value was calculated as the proportion of true positive lesions for AI or a reader when compared to another reader used as reference, divided by true positive plus false positive lesions when compared to the same reference reader. For all analyses, each of the three readers was alternately used as a reference and pairwise compared to either AI or another reader, and the average and range of all pairwise combinations were calculated. The specificity and negative predictive value cannot be calculated on a lesion basis since it is not possible to calculate non-malignant lesions not detected by either method.

## Results

**Fig. 2:**
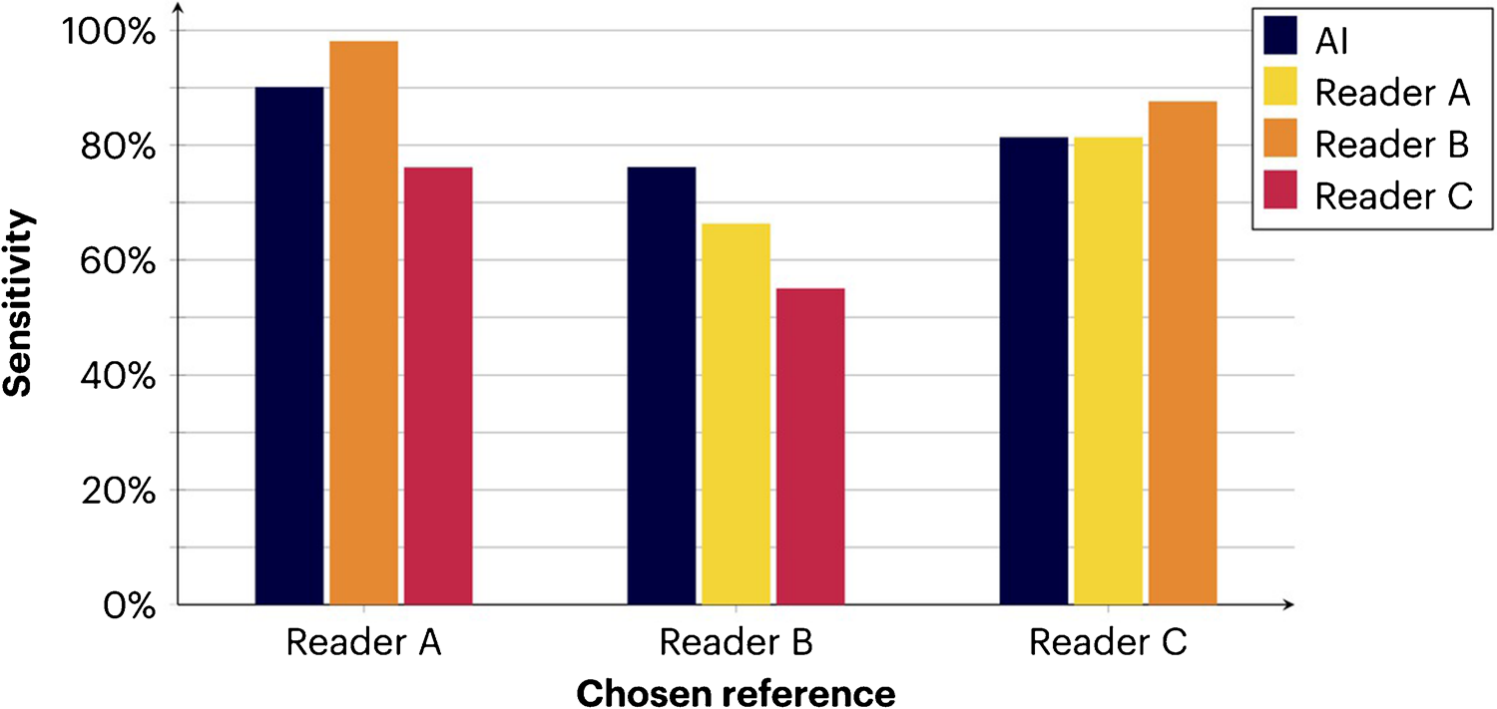
Sensitivity of the AI model and between readers when using reader A, reader B, and reader C as a reference, respectively

The AI model had 76–90% sensitivity (average 82%) for the detection of suspected pelvic lymph node metastasis on a lesion level when considering each of the three readers as a reference (Table 1, Fig. 2). When each reader was alternately used as a reference and tested against the other readers, the average sensitivity was 77% (range 55–98% depending on which of the readers that was used as a reference vs. test). Thus, the sensitivity for the AI model was well in the inter-reader range. Table 1 also shows the number of false positive lesions and the total number of false negative lesions detected by the AI model with each reader as a reference. As shown, the number of false positives on a patient level was low (mean 1.8 false positives per patient).

**Table 1:** True and false positives (*TP/FP*), false negatives (*FN*), sensitivity, and positive predictive value (*PPV*). The number of true/false positives is shown for the whole test group and per patient for AI vs. reader and reader vs. reader (average and range; where one reader at a time was used as a reference). Sensitivity and PPV are similarly shown as the average and range when one reader at a time was used as a reference

*n* = 50 patients	**AI vs. reader**	**Reader vs. reader**
TP		
-Total -Per patient	46.0 (39.0–54.0) 0.9 (0.8–1.1)	42.3 (38.0–49.0) 0.9 (0.8–1.0)
FP		
-Total -Per patient	91.3 (80.0–102.0) 1.8 (1.6–2.0)	14.0 (1.0–32.0) 0.3 (0.02–0.6)
FN	10.3 (5.0–17.0)	14.0 (1.0–32.0)
Sensitivity (%)	82.4 (76.1–90.0)	77.3 (54.9–98.0)
PPV (%)	33.6 (27.7–40.3)	77.6 (56.8–97.9)

### Detailed analysis of false positive and negative lesions with reader A as a reference

False positive lesions were most often found in pelvic lymph nodes (not marked as suspected metastases by any reader) or in the gastrointestinal tract. A few false positive lesions were also found in areas of high PSMA uptake located in the prostate, seminal vesicles, or bone. The detailed analysis for reader A showed a total of 92 uptakes detected by the AI model but not by reader A (false positives), of which 13 were located in a suspected tumour in prostate/seminal vesicles, 35 were in pelvic lymph nodes not marked by the reader, 35 were in the gastrointestinal tract, 1 was in a suspected bone metastasis, and 8 were in other locations.

**Fig. 3:**
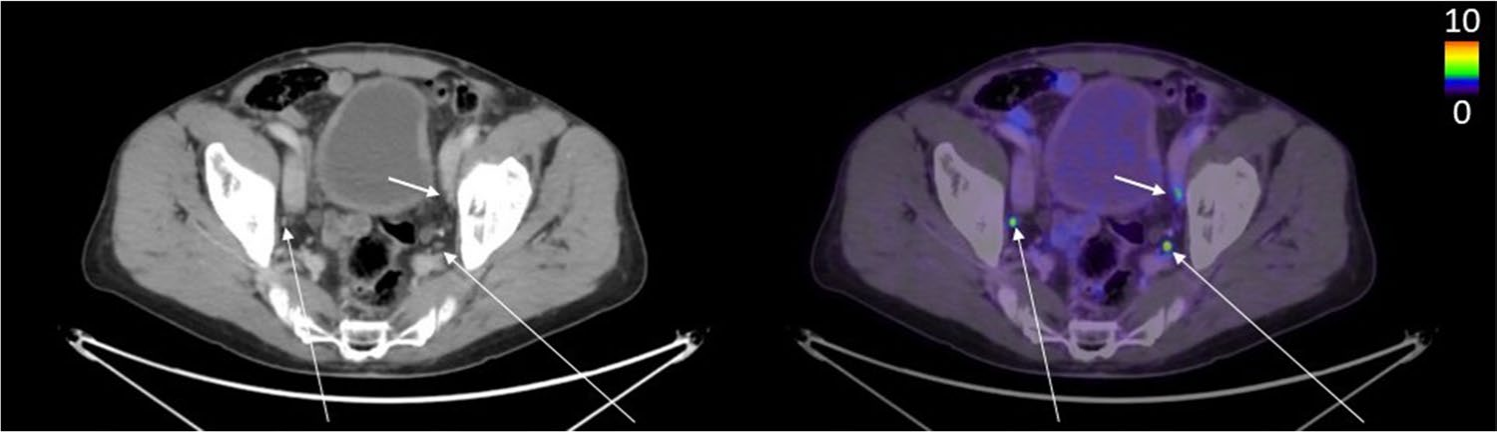
The two long arrows indicate lymph node metastases detected by all readers and the AI model (true positives). The short arrow shows a lymph node marked as a metastasis by reader B (false negative when reader B is ground truth) but not by reader A, reader C, or the AI model

**Fig. 4:**
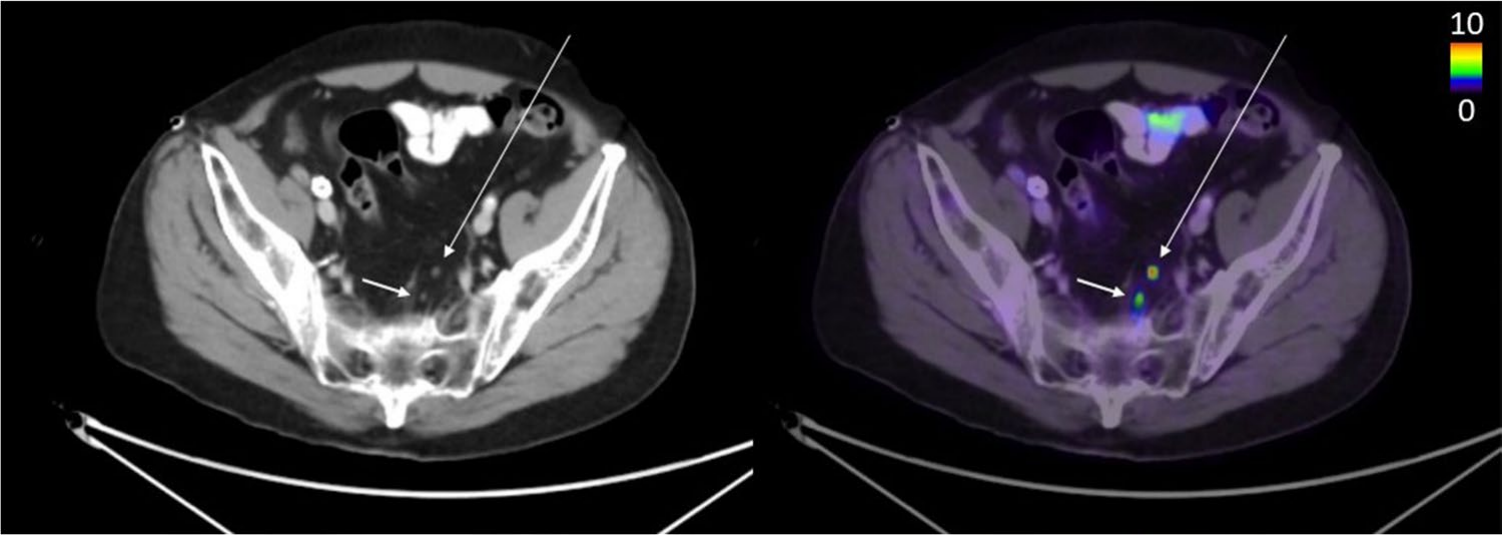
The long arrow shows a lymph node metastasis detected by all readers and the AI model (true positive). The short arrow shows a lymph node metastasis marked by all readers but not detected by the AI model (false negative)

**Fig. 5:**
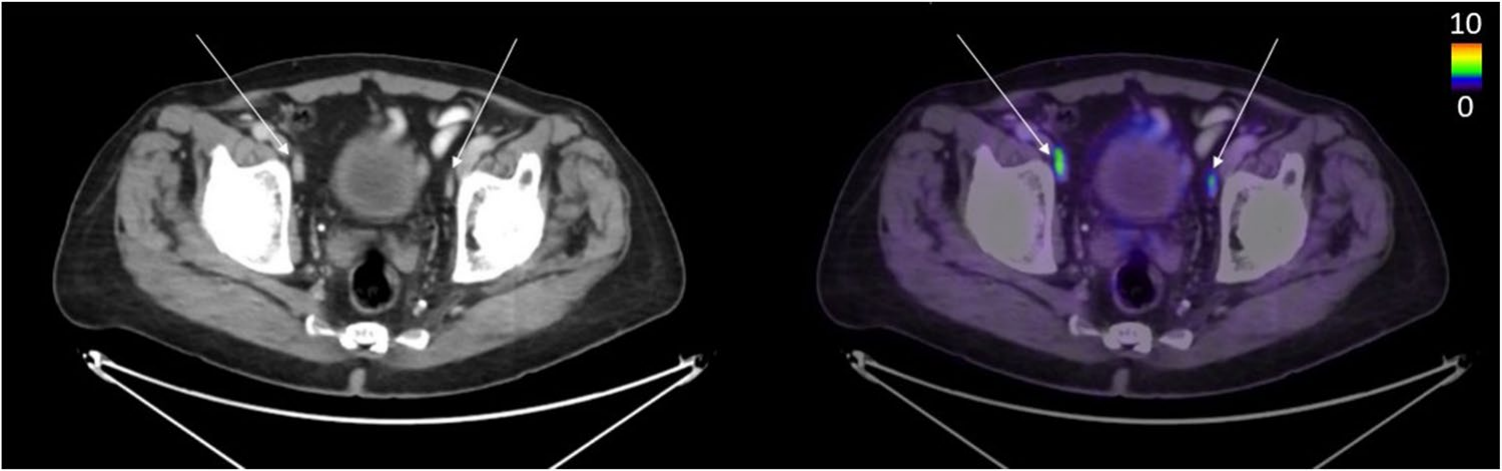
The arrows show lymph nodes detected as suspected metastases by the AI model and by reader B (true positive when reader B is ground truth), but not by readers A and C (false positives when these readers are ground truth; regarded as unspecific uptake by these readers)

False negative lesions (lesions marked as lymph node metastases by a reader that were missed by the AI model) were often found in presacral/mesorectal lymph nodes. For example, of the 5 false negative metastases when considering reader A as ground truth, 3 were located in the presacral area. Figures 3, 4, and 5 show representative findings of true positive, false negative, and false positive lymph node metastasis. The AI tool has been made freely available for researchers at www.recomia.org or by e-mailing contact@recomia.org.

## Discussion

In this study, we developed and validated an AI-based method for the detection of pelvic lymph node metastases in [^18^F]PSMA-1007 PET-CT scans in patients with high-risk prostate cancer who were referred for initial staging. The AI model had rather high sensitivity (average 82%, range 76–90% depending on which of the three readers that was used as a reference). The sensitivity of the AI model was well in the inter-observer range for the three readers (average 77%, range 55–98% depending on which of the three readers that was used as a reference). The AI model had an average of only 1.8 false positive lesions per patient.

Current imaging techniques, including PET-CT, are challenged by time-consuming manual analysis and issues with inter-reader agreement. Despite attempts to standardize reporting of PSMA PET/CT scans, there is still intra- and inter-reader disagreement, even from single centres [8, 9]. The readers in this study were well experienced but despite the fact, inter-reader agreement was not perfect. The results support the need for a tool to help reduce this problem. In many countries, there is also a lack of trained nuclear medicine physicians or radiologists to analyse the scans. Furthermore, it has been shown that tumour burden and the corresponding therapeutic radioligand dose received by the tumour correlate with the treatment response of PSMA-directed radioligand therapy [21].

PSMA radionuclide therapy is typically offered to patients with multiple metastases where it is impractical and time consuming to manually delineate all lesions. Therefore, there is a need for fast, automated, objective, and reproducible image analysis. AI tools offer an interesting opportunity to improve reproducibility as well as reading times for PET-CT analysis.

A high number of false positive detections has been a major clinical limitation for clinical AI tools in mammography and lung imaging, as well as for PSMA PET-CT. It can be time consuming for physicians to review and dismiss a large number of false positive detections, and there is also a risk that it could lead to decreased specificity [22]. Therefore, the low number of false AI detections of pelvic lymph nodes in this study (1.8 per patient) was encouraging.

A recent paper by Johnsson et al. [10] found a sensitivity of just over 90% for automated detection of pelvic lymph node metastases for [^18^F]DCFPyL PET-CT imaging. However, on average, there were 19.5 false positive pelvic lesions per patient (compared with 1.8 in our study) and 90.5 false positive lesions per patient when extra-pelvic lymph nodes were also detected. Another study by Zhao et al. [11] also aimed to develop an AI-based method for the detection of pelvic bone and lymph node lesions on [^68^ Ga]Ga-PSMA-11 PET-CT scans and found 90% sensitivity for the detection of pelvic lymph node metastases. They did not state the number of false positive uptakes. They only considered the pelvic region of the scans (small field of view) and used a patient cohort with considerably more metastases than ours, making the two studies difficult to compare.

One might wonder whether complicated methods such as CNNs are needed to detect metastases accurately or whether it is sufficient to use simple thresholds. Recent studies have used SUV thresholds above 3.0 or 4.3 for detecting lesions [12, 13]. Johnsson et al. [10] investigated these thresholds in a patient cohort and found a significant drop in sensitivity. For comparison, we applied a SUV threshold of 3.0 to our material, which resulted in a very high number of false positive lesions that the readers did not select, on average 19.9 per patient (range 19.6–20.1 depending on which reader was used as reference). This is over 10 times more compared to our method which supports the application of AI. It is also well known that the use of different PET-CT scanners, acquisition protocols, and reconstruction algorithms results in different SUV, especially in small lesions [23], which may limit the usefulness of SUV thresholds.

The limitations of this study include the relatively small number of PET-CT scans and that all studies and readers came from a single centre. We also did not have any external validation, such as verification of lymph node metastases by histopathology. Only 21 of the 211 patients in the study underwent extended pelvic lymph node dissection after the PET-CT scan, 6 of which in the test group. The study was also based on only one of several available PSMA tracers. The performance of the AI tool needs to be evaluated in PET-CT scans based on other PSMA tracers as well as on studies performed at other hospitals. In one of the next steps, an AI model will be trained to detect lymph node metastases outside the pelvis as well as primary tumours and bone metastases. Furthermore, it will include PET-CT scans acquired at both 1 and 2 h after injection of the radiopharmaceutical.

## Conclusion

This study has shown that AI can obtain a sensitivity that is well within the inter-observer range with a reasonable number of false positives per patient. The difficulty in achieving high inter-observer sensitivity emphasizes the need for automated methods, especially for new or unusual tracers. On the road to qualifying AI tools for clinical use, independent validation is critical for allowing performance to be assessed in studies from different patients and clinical settings. Thus, we have made our AI tool freely available to other researchers at www.recomia.org or by e-mailing contact@recomia.org.

## Data Availability

The AI method is freely available for research at www.recomia.org.
